# Novel celastrol derivatives inhibit the growth of hepatocellular carcinoma patient-derived xenografts

**DOI:** 10.18632/oncotarget.2171

**Published:** 2014-07-10

**Authors:** Wei Wei, Song Wu, Xiaolin Wang, Chris Kin-Wai Sun, Xiaoyang Yang, Xinrui Yan, Mei-Sze Chua, Samuel So

**Affiliations:** ^1^ Asian Liver Center, Department of Surgery, Stanford University School of Medicine, Stanford, CA; ^2^ School of Pharmaceutical Sciences, Wuhan University, Wuhan P. R. China; ^3^ Department of Radiology, Molecular Imaging Program at Stanford University, Stanford, CA

**Keywords:** celastrol derivatives, molecular chaperone, targeted therapy

## Abstract

The molecular co-chaperone CDC37 is over-expressed in hepatocellular carcinoma (HCC) cells, where it functions with HSP90 to regulate the activity of protein kinases in multiple oncogenic signaling pathways that contribute towards hepatocarcinogenesis. Disruption of these signaling pathways *via* inhibition of HSP90/CDC37 interaction is therefore a rational therapeutic approach. We evaluated the anti-tumor effects of celastrol, pristimerin, and two novel derivatives (cel-D2, and cel-D7) on HCC cell lines *in vitro* and on orthotopic HCC patient-derived xenografts *in vivo*. All four compounds preferentially inhibited viability of HCC cells *in vitro*, and significantly inhibited the growth of three orthotopic HCC patient-derived xenografts *in vivo*; with the novel derivatives cel-D2 and cel-D7 exhibiting lower toxicity. All four compounds also induced cell apoptosis; and promoted degradation and inhibited phosphorylation of protein kinases in the Raf/MEK/ERK and PI3K/AKT/mTOR signaling pathways. We demonstrated that HSP90/CDC37 antagonists are potentially broad spectrum agents that might be beneficial for treating the heterogeneous subtypes of HCC, either as monotherapy, or in combination with other chemotherapeutic agents.

## INTRODUCTION

Hepatocellular carcinoma (HCC), the most common adult liver malignancy, is the seventh most common cancer and the second most frequent cause of cancer-related death worldwide [1]. Most of the burden (80%) of HCC is borne in the developing world, such as Eastern and Southeast Asia and sub-Saharan Africa, where the dominant risk factor is chronic infection with hepatitis B virus (HBV), together with exposure with aflatoxin B1. In developed countries, including North America, Europe, and Japan, the dominant risk factor is chronic infection with hepatitis C virus [2].

HCC has a poor prognosis, partly due to late diagnosis of the disease and lack of effective therapeutic options [3]. Most patients remain asymptomatic until the disease is advanced. Although more than 50 drugs that target different biomarkers or signaling pathways are in clinical trials for HCC treatment [4], as yet there is no therapeutic agent superior to sorafenib, which was FDA approved as the standard of care for advanced HCC [5]. Sorafenib is a tyrosine kinase inhibitor that inhibits cell proliferation *via* disrupting the Raf/MEK/ERK pathway, and that blocks angiogenesis *via* inactivating the functions of VEGFR and other growth factor receptors [6]. However, sorafenib only modestly improves overall survival of HCC patients by less than three months [5]. Due to recent emergence of resistance to sorafenib (7), second-line therapies targeting other key signaling pathways in HCC, such as the EGFR, WNT, and PI3K-AKT-mTOR pathways [7], are highly desirable.

The chaperone-kinome pathway is particularly attractive as a therapeutic target in cancer [8, 9]. In this pathway, the heat shock protein 90 (HSP90) cooperates with its molecular co-chaperone CDC37 to regulate the folding, maturation, stabilization, and phosphorylation of a wide array of protein kinases, which are important mediators of signal transduction and cell growth in human cancers [10]. HSP90 has been recognized as a key facilitator of oncogene addiction and a promising therapeutic target in cancers [11], with several HSP90 inhibitors in preclinical and clinical evaluation for cancer therapy [12, 13]. Current HSP90 inhibitors interact with the N-terminal ATP-binding pocket and block ATP binding to stop the chaperone cycle, thereby leading to client protein degradation [11]. Despite encouraging preclinical efficacy of HSP90 inhibitors, clinical translation is likely limited due to the activation of the heat shock transcription factor HSF1 [14], which induces the expression of HSP70 and HSP23 to protect tumor cells from apoptosis [13-15].

On the contrary, targeting of the co-chaperone CDC37 have several advantages over targeting HSP90 [10]. Firstly, it obviates the undesirable induction of the anti-apoptotic heat shock response seen with HSP90 inhibition, and additionally inhibits the HSF-1 activity and HSP70 expression [16, 17]. Secondly, CDC37 is the key permissive factor in cell transformation caused by oncogenic protein kinases [8, 10, 18]. Crystal structure studies suggest that the central segment of CDC37 associates with the N-terminal ATPase domain of HSP90 [19], whereas the N-terminal segment of CDC37 associates with its client protein kinases [20]. Many of its client protein kinases are dysregulated or activated in HCC, including Cdk4, EGFR, AKT, MEK1/2 and Raf family proteins [21-24]. Thirdly, over-expression of CDC37 has been reported in various cancers such as prostate cancer [25], multiple myeloma [26], anaplastic large cell lymphoma [27], acute myelocytic leukaemia [28], and HCC [29]. In prostate epithelial cells, the aberrant expression of CDC37 contributes towards carcinogenesis by stabilizing and activating its client protein kinases, thereby promoting cell proliferation and survival [30]. Lastly, CDC37 expression is increased in proliferating tissues and is heavily expressed in certain cancers (due to the increased need of over-expressed protein kinases that mediate their growth); whereas most normal tissues do not proliferate or appear to require CDC37 [10, 31]. This specificity of CDC37 for malignant cells potentially offers a greater therapeutic window for CDC37-based therapeutics.

Given the contributory roles of multiple protein kinases in hepatocarcinogenesis, we hypothesized that disrupting the HSP90 and CDC37 chaperone complex may achieve anti-tumor effects in HCC, by inducing degradation and inhibiting phosphorylation of their client proteins kinases [16, 32, 33]. We therefore evaluated the recently identified HSP90/CDC37 antagonist, celastrol [34], for its anti-tumor activity in HCC cell lines and patient-derived xenografts. We additionally synthesized three derivatives of celastrol and compared their safety and anti-tumor activity profiles in HCC patient-derived xenografts, providing a clinically relevant model for evaluating the performance of these potential therapeutic compounds.

## RESULTS

### Synthesis and characterization of celastrol derivatives that disrupt HSP90/CDC37 complexes

The chemical structures of celastrol and its derivatives are shown in Table 1. All chemical structures were confirmed by analyses using ^1^H (Supplementary Fig. 1 for cel-D2; Supplementary Fig. 2 for cel-D7) and ^13^C NMR (Supplementary Fig. 3 for cel-D2; Supplementary Fig.4 for cel-D7), and high resolution mass spectrometry (Supplementary Fig. 5 for cel-D2; Supplementary Fig. 6 for cel-D7).

**Table 1:** Structure and activity of celastrol and its derivatives

Compound	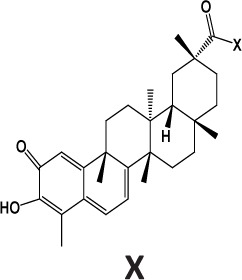	IC_50_ in HCC cell lines (μM)			IC_50_ in normal hepatocytes (μM)
		HepG2	Huh7	Hep3B	
celastrol	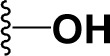	1.22±0.12	1.07±0.13	0.30±0.08	2.06−3.08
pristimerin	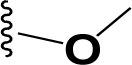	1.7±0.21	0.68±0.05	0.85±0.08	3.87−5.33
cel-D2	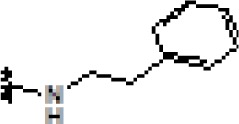	3.58±0.32	1.04±0.05	1.06±0.10	5.9−16.8
cel-D7	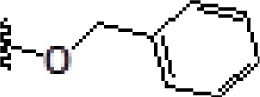	4.26±0.23	2.15±0.14	2.77±0.32	15.66−23.95

To confirm that these derivatives of celastrol retain the ability to disrupt HSP90/CDC37 interaction, we first did Western blot analysis of HSP90 after immunoprecipitation of CDC37 from HepG2 cell lysates, which demonstrated that immunoprecipitation of CDC37 pulled down HSP90 as expected. Treatment of HepG2 cells for 6 hours with celastrol or its three derivatives at 5 μM each decreased the amount of HSP90 in the immunoprecipitated CDC37 complex. Our results showed that HSP90 and CDC37 formed a complex *in vitro* and that all four chemicals disrupted their direct interaction.

**Fig. 1:**
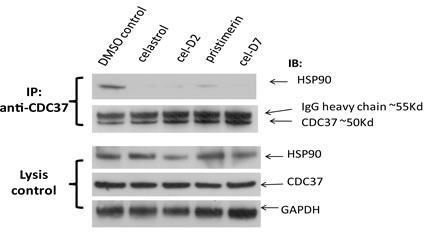
Celastrol and its derivatives disrupt HSP90/CDC37 interaction in HCC cells HepG2 cells were incubated with 5 μM of each compound and the same volume of DMSO as negative control for 6 hours. HSP90/CDC37 complex was then pulled down by anti-CDC37 antibody in the cell lysates. Anti-HSP90 antibody was used to detect the HSP90 protein in the complex. The lysates were used to detect HSP90, CDC37, and GAPDH (loading control).

### Celastrol and its derivatives preferentially inhibited viability of HCC cells compared to normal hepatocytes

**Fig. 2:**
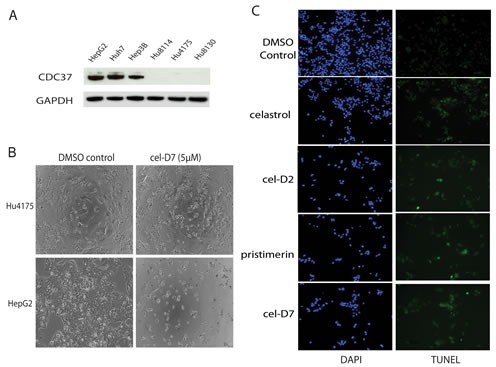
Celastrol and its derivatives are preferentially inhibited viability of HCC cells compared to normal hepatocytes A). CDC37 and GAPDH (loading control) expression were determined by Western Blot using specific antibodies in HCC cells (HepG2, Huh7, and Hep3B) and normal hepatocytes (Hu8114, Hu4175, and Hu8130). B). Phase-contrast microscopic examination of the effect of cel-D7 (5 μM) on HepG2 cells and normal hepatocytes Hu4175. C). Celastrol and its derivatives (5 μM each) induced apoptosis in Huh7 cells after 6 hours treatment. Cells were stained with TUNEL and DAPI as described under Materials and Methods to detect for apoptotic cells. Fluorescence labeling was visualized and photographed at 100x magnification.

To test our hypothesis that HSP90/CDC37 antagonists are feasible anti-tumor agents in the treatment of HCC, we first tested their selective cytotoxicity against HCC cells (HepG2, Huh7, and Hep3B) compared to normal hepatocytes (Hu8114, Hu4175, and Hu8130, obtained from three donors with non-diseased liver). Western blot analysis confirmed that only HCC cells express high levels of CDC37, whereas normal hepatocytes express undetectable levels of CDC37 (Fig. 2A). Accordingly, treatment of these HCC cell lines and normal hepatocytes with celastrol or its derivatives for 3 days showed greater inhibition of cell viability in HCC cell lines compared to normal hepatocytes, with several folds lower IC_50_s in HCC cell lines than in normal hepatocytes for each compound (Table 1; Supplementary Fig. 7). Light microscopic examination demonstrated greater toxicity towards HCC cells compared to normal hepatocytes (representative images for treatment with cel-D7 are shown in Fig. 2B). Each of these compounds also induced apoptosis in HCC cell lines (representative images for Huh7 cells are shown in Fig. 2C). Our data indicate that modification of the carboxylic acid group of celastrol retained HSP90/CDC37 antagonist activity, as well as anti-tumor activity in HCC cell lines. Notably, celastrol and pristimerin, both with non-aromatic substituents, show greatest activity against HCC cells, and are also considerably more toxic against normal hepatocytes. Cel-D2 and cel-D7, with aromatic phenyl substituents, exhibited reduced activity against HCC cells; with cel-D7 being the least toxic against normal hepatocytes.

### Celastrol and its derivatives induced degradation and inhibited phosphorylation of HSP90/CDC37 client protein kinases in HCC cell lines

**Fig. 3:**
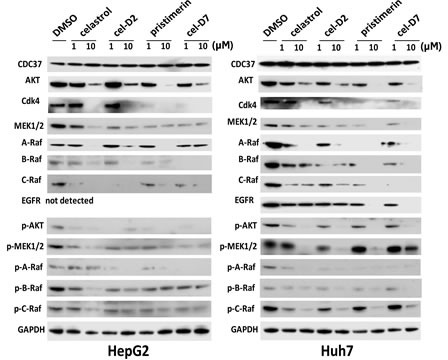
Celastrol and its derivatives induced degradation and inhibited phosphorylation of HSP90/CDC37 client proteins in HCC cell lines HepG2 and Huh7 cells were incubated for 6 hours with each compound (at 1 or 10 μM) and CDC37, HSP90/CDC37 client proteins and GAPDH (loading control) levels were determined by Western blotting using specific antibodies.

To study the molecular events resulting from treatment with celastrol and its derivatives, we detected the levels and phosphorylation status of several HSP90/CDC37 client protein kinases that are known to be highly activated in HCC, including the Raf family proteins, AKT, MEK1/2, CDK4, and EGFR. Treatment of HepG2, Huh7, and Hep3B cells with celastrol and its derivatives for 6 hours reduced the protein levels and phosphorylation levels of all the HSP90/CDC37 client protein kinases studied, compared to DMSO control (Fig. 3 and Supplementary Fig. 8). These effects were dose-dependent, with 10 μM concentration of each compound causing greater reductions in expression and phosphorylation levels for almost all protein kinases compared to treatment with 1 μM of each compound. EGFR was not detected in HepG2 cells, but showed decreased levels after treatment with all compounds in Huh7 cells (with dose-dependence seen only with pristimerin and cel-D7). CDC37 levels did not change after treatment with any compound.

### Celastrol and its derivatives inhibited growth of orthotopic HCC patient-derived xenografts

**Fig. 4:**
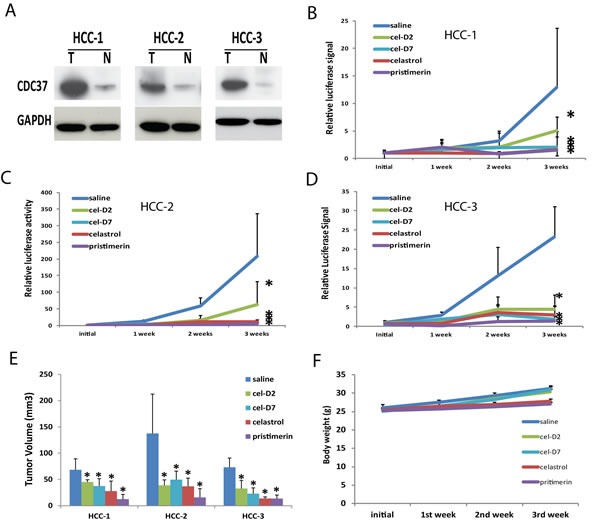
Celastrol and its derivatives inhibited growth of orthotopic HCC patient-derived xenografts A). CDC37 and GAPDH (loading control) protein expressions in the tumor (T) and matched non-tumor liver (N) tissues of three HCC patients were determined by Western Blot. B, C, D). Growth curves based on bioluminescence signals for HCC-1, HCC-2, and HCC-3 during the 3-week treatment period for each compound and saline control. *P < 0.05 for each compound *vs.* saline control group (n=5 each). E). Tumor volumes of HCC-1, HCC-2, and HCC-3 xenografts during the 3-week treatment period for each compound and saline control. *P < 0.05 for each compound *vs.* saline control group (n=5 each). F). Representative body weight curve of mice bearing HCC-3 xenografts during the 3-week treatment period with all four compounds.

We next evaluated the anti-tumor effects of celastrol and its derivatives in orthotopic HCC patient-derived xenografts. Tumors from three HCC patients (HCC-1, HCC-2, and HCC-3) were confirmed to express high levels of CDC37 compared to their matched non-tumor liver tissues (Fig. 4A). Preliminary limited toxicity studies in NSG mice suggested the maximum tolerate dose was 4 mg/Kg for celastrol; 1 mg/Kg for pristimerin; 8 mg/Kg for cel-D2; and 8 mg/Kg for cel-D7. At these respective doses, all four compounds did not result in significant toxicity or any noticeable discomfort to the mice, suggesting that these doses were well tolerated. We therefore used these doses for treatment, which was initiated within one week after orthotopic transplant of the xenografts stably expressing the luciferase reporter gene.

Based on weekly monitoring of luciferase signal *in vivo*, all four compounds significantly inhibited growth of all three orthotopic HCC xenografts (compared to saline treated controls) at the end of the 3-weeks treatment period (Fig. 4B-D; Supplementary Fig. 9). Measurement of final tumor volumes at the end of the treatment period consistently showed that all compounds caused significant reductions in tumor volumes in all three orthotopic HCC xenografts (when each treatment group is separately compared to saline control group) (Fig. 4E; P < 0.05). On average across all three HCC xenografts, celastrol reduced tumor volumes by 2-5 folds; pristimerin by 5-7 folds; cel-D2 by 1.5-3.5 folds; and cel-D7 by 1.8-3.2 folds. Our *in vivo* data were consistent with our *in vitro* data, with celastrol and pristimerin showing greater anti-tumor activity than cel-D2 and cel-D7. Of note, tumor mass was absent from one of five mice in the group with HCC-1 xenografts after celastrol treatment, and from one of five mice in all three groups with HCC-1, HCC-2, or HCC-3 xenografts after pristimerin treatment.

*In vivo* toxicology analysis revealed that celastrol and pristimerin caused slight decreases in the body weight of treated mice, whereas cel-D2 and cel-D7 did not affect body weight of treated mice (compared to saline treated controls) (Fig. 4F and Supplementary Table 3A). In addition, white blood cells were elevated by about 2.5-folds in the celastrol treatment group (16.32 ± 3.23 K/μL) as compared to the saline control group (6.34 ± 2.34 K/μL). Correspondingly, mice treated with celastrol have enlarged spleens (Supplementary Table 3A). We also noted that pristimerin treatment significantly elevated the blood AST and ALT level as compared to saline control group (Supplementary Table 3B), suggesting that pristimerin may negatively impact liver functions. However, cel-D2 and cel-D7 treatments did not result in any significant changes in body weight or blood chemistries. These *in vivo* observations were consistent with our *in vitro* data that celastrol and pristimerin are more toxic to normal hepatocytes.

### Celastrol and its derivatives induced apoptosis and degradation of HSP90/CDC37 associated client protein kinases in orthotopic HCC patient-derived xenografts

**Fig. 5:**
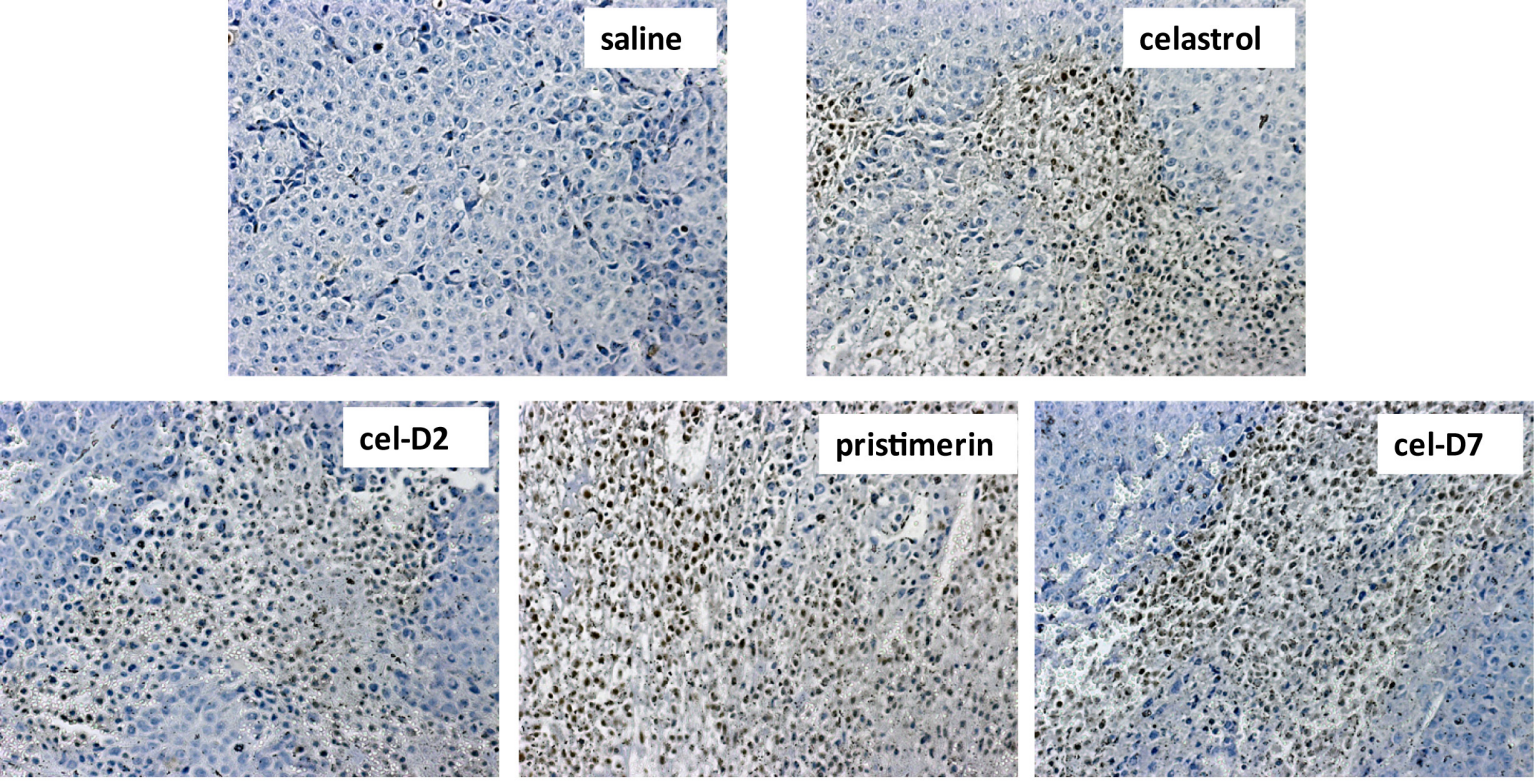
Celastrol and its derivatives induced apoptosis (TUNEL Assay) in orthotopic HCC patient-derived xenografts Representative images are shown for HCC-3 xeongrafts after 3 weeks treatment of each compound and saline control (200x magnification). Apoptotic cells are defined by cells with brown nucleic staining.

**Fig. 6:**
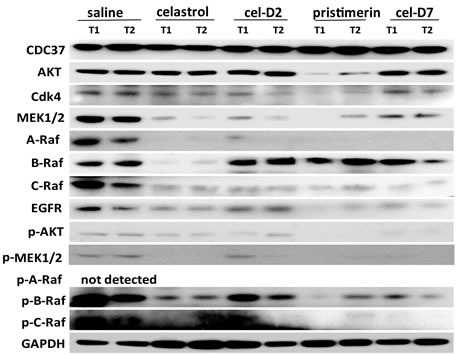
Celastrol and its derivatives induced degradation and inhibited phosphorylation of HSP90/CDC37 client proteins in orthotopic HCC patient-derived xenografts Representative Western Blot results are shown for client proteins and their phosphorylation levels in two random tumors (T1 and T2) of HCC-3 xenografts after 3 weeks of treatment with each compound and saline control. GAPDH was used as a loading control.

Similar to our *in vitro* observations with HCC cell lines, all four compounds induced cell apoptosis in all the orthotopic HCC xenografts when harvested tumors were analyzed by TUNEL staining (representative images for HCC-3 xenografts are shown in Fig. 5). Celastrol and pristimerin showed more extensive staining of apoptotic cells than cel-D2 and cel-D7, consistent with the greater anti-tumor activities observed with the former two compounds. Western blot analysis of HSP90/CDC37 client protein kinases demonstrated that all four compounds induced degradation and inhibited phosphorylation of most of the studied kinases (representative images for HCC-3 are shown in Fig. 6). Pristimerin in particular showed strongest effects on AKT, MEK1/2 and EGFR, whereas celastrol showed strongest effect on B-Raf. CDC37 expression in these xenografts were maintained (compared to original HCC tumor levels), and expression levels were unaffected by treatment with all four compounds.

## DISCUSSION

HSP90 and its co-chaperone CDC37 are key factors in the chaperone-kinome pathway that is recognized to play permissive roles in the oncogenesis of multiple types of tumors, including HCC [10]. The inhibition of HSP90 or CDC37 alone (by RNA interference or by small molecule inhibitors of HSP90) has shown encouraging anti-tumor effects in cell-based studies of multiple tumors, which are associated with enhanced degradation and decreased phosphorylation of oncogenic HSP90/CDC37 client protein kinases [11, 16, 17, 33]. Based on the established role of these client protein kinases in hepatocarcinogenesis, we hypothesized that the direct disruption of HSP90 interaction with CDC37 by the small molecule celastrol would achieve desirable anti-tumor effects. Indeed, celastrol and three of its derivatives were successfully shown to disrupt HSP90 and CDC37 interaction in HCC cells; to inhibit the growth of secondary HCC cell lines *in vitro*; and to inhibit the growth of orthotopic HCC patient-derived xenografts *in vivo*.

The observed anti-tumor activities of celastrol and its derivatives both *in vitro* and *in vivo* promoted degradation and decreased phosphorylation of protein kinases that are known to be highly activated in HCC cells, such as Raf family proteins, AKT, MEK1/2, CDK4, and EGFR. This ability to simultaneously disrupt multiple oncogenic signaling pathways suggests that these HSP90/CDC37 antagonists are potentially broad spectrum inhibitors that would be beneficial for treating the heterogeneous subtypes of HCC. In particular, the Raf/MEK/ERK and PI3K/AKT/mTOR pathways are both critically involved in hepatocarcinogenesis [35-38]. Raf/MEK/ERK signaling is activated in more than 50% of HCC [39], whereas PI3K/AKT/mTOR signaling is activated in about 40-50% of HCC [23, 35, 40]. The inhibition of both these pathways by molecules specifically targeting each pathway have successfully suppressed HCC growth [35, 36, 38]. Our results suggest that HSP90/CDC37 antagonists can effectively inhibit both these (and other) pathways simultaneously, offering the benefit of treating a larger percentage of HCC patients without the need to administer chemotherapeutic cocktails that would also increase side effects.

An additional benefit resulting from simultaneous inhibition of a wide array of oncogenic kinase pathway is the potential to help overcome drug resistance that are often associated with the activation of one or more of these pathways. For example, acquired drug resistance to sorafenib (targeting angiogenesis and Raf/MEK/ERK signaling pathways) have been attributed to the activation of PI3K/AKT and EGFR [41, 42]; adaptive drug resistance to EGFR-targeted therapies have been associated with activation of PI3K/AKT pathway [43]; whereas therapies targeting AKT/mTOR leads to Raf/MEK/ERK pathway activation through a PI3K-dependent feedback loop [44]. Thus, the use of HSP90/CDC37 antagonists either by themselves or in combination therapy with other pathway-specific inhibitors may potentially circumvent the development of acquired drug resistance by inhibiting multiple pathways simultaneously, and at the same time increase drug sensitivity. This is particularly beneficial for HCC, which is highly resistant to currently used chemotherapeutic drugs, making successful treatment a clinical challenge.

In our attempt to develop new and more efficacious derivatives of celastrol, we synthesized a total of seven derivatives (cel-D1 to cel-D7, with cel-D5 being pristimerin) by introducing different substituents (aromatic or non-aromatic) via amide or ester bond to the chemically active carboxylic acid group of celastrol. The structures and IC_50_s of all seven derivatives are shown in Supplementary Table 1. We selected celastrol, pristimerin, cel-D2 and cel-D7 for further studies based on their greater preferential activities against HCC cells compared to normal hepatocytes. Among them, our data suggest that celastrol and pristimerin have greater anti-tumor efficacy but also greater toxicity both *in vitro* and *in vivo* (observed as lower IC_50_ to normal hepatocytes *in vitro* and greater weight loss *in vivo*); whereas cel-D2 and cel-D7 have slightly reduced anti-tumor efficacy and reduced toxicity (observed as higher IC_50_ to normal hepatocytes *in vitro* and absence of weight loss *in vivo*). Celastrol treatment also caused enlarged spleen and elevated white blood cells; whereas pristimerin treatment caused significant liver impairment (elevated AST and ALT level). At the molecular level, celastrol and pristimerin showed greatest inhibitory effects on the client protein kinases, which may underlie their greater apoptotic effects to both malignant and normal hepatocytes. Our limited structure-activity-relationship (SAR) study suggests that modifications of the carboxylic acid group of celastrol do not drastically affect their ability to disrupt HSP90/CDC37 inhibition; however, derivatives with aromatic phenyl substituents (cel-D2 and cel-D7) appear to induce less marked apoptosis and may therefore be less toxic to normal cells compared to derivatives with non-aromatic alkyl groups. The clinical translation of celastrol and pristimerin may be limited due to their toxicity, and further pharmacokinetics and pharmacodynamics evaluation of cel-D2 and cel-D7 are warranted before determining their suitability for use in human patients. It is likely that detailed SAR studies may lead to the identification of other derivatives which may have an improved therapeutic window.

The use of orthotopic patient-derived xenografts in our study provides a fairly high predictive value of the response of HCC patients to celastrol and its derivatives. The human origin of these xenografts more accurately reflect the response rates; in fact, responses to chemotherapy in patient-derived xenografts have been reported to resemble the response rates of monotherapy in clinical trials [45]. Orthotopic models have also been shown to be more predictive of a patient's response to chemotherapy [46]. Our xenografts are established from HCC patients with over-expression of CDC37, which we confirmed to be maintained in the xenografts. In our experience, we observed that not all HCC patient tumors can establish xenografts in immunocompromised mice; of those that successfully engrafted, the expression of CDC37 and its related protein kinases were found to be consistently high, suggesting that these proteins and their associated signaling pathways are critical factors in allowing engraftment. The maintenance of these molecular features in our patient-derived xenografts is especially relevant in the evaluation of the efficacy of celastrol and its derivatives. Thus, in future preclinical or clinical studies of HSP90/CDC37 antagonists, it is recommended to screen tumor models or patients for the expression of CDC37 and its client protein kinases before considering treatment with these agents.

In summary, we demonstrated that HSP90/CDC37 antagonists are promising agents for the treatment of HCC, which are typically resistant to standard chemotherapeutic agents. They are potentially broad spectrum agents, being able to simultaneously disrupt multiple oncogenic pathways that are critical in development of the heterogeneous subtypes of HCC. Thus, HSP90/CDC37 antagonists may be effective as monotherapy or as combination therapy with other conventional agents, with the additional potential to sensitize HCC cells and circumvent the development of acquired resistance to these agents. Our data suggest that celastrol in itself might be too toxic for clinical use, but that two of its derivatives with reduced toxicity might be considered for further preclinical evaluation or structure-activity optimization. We conclude that targeting the chaperone-kinome pathway is a promising approach for the treatment of HCC.

## MATERIALS AND METHODS

### Synthesis of celastrol derivatives

Celastrol and pristimerin were purchased from Sigma-Aldrich (St. Louis, MO). Two other celastrol derivatives, cel-D2 and cel-D7, were synthesized using celastrol as the starting material, using chemicals purchased from Sigma-Aldrich. Synthetic schemes for cel-D2 and cel-D7 are shown in Supplementary Fig. 10. The ^1^H and ^13^C NMR spectra were obtained using the Varian 300 MHz or 400 MHz magnetic resonance spectrometer. High resolution mass spectrometric (MS) analyses of the compounds were performed at the Mass Spectrometry Facility at Stanford University. For cel-D2, celastrol (20.5 mg, 0.045 mmol) was dissolved in dimethylformamide (2 mL), DIPEA (20 μL, 0.12 mmol) and PyBOP (50 mg, 0.096 mmol) were then added into the solution, followed by 2-phenylethylamine (10 μL, 0.079 mmol). After stirring for 24 hours at room temperature, deionized water (15 mL) was added and the reaction mixture was extracted by ethyl acetate (3 × 15 mL). The combined organic extracts were dried over MgSO4, filtered and concentrated *via* a rotary evaporator to yield a dark red oil. Reversed phase (RP)-HPLC (Dionex HPLC System; Dionex Corporation, Sunnyvale, CA) using a C18 column (Phenomenax, 5 μm, 4.6 × 250 mm or Dionex, 5 μm, 21.2 × 250 mm) and an acetonitrile-water gradient mobile phase (flow of 1 or 12 mL/minutes) afforded cel-D2 as an orange solid (17.2 mg, 68.0% yield), with m/z 554.3621 (M+H).

For cel-D7, celastrol (20.2 mg, 0.045 mmol) was dissolved in dimethylformamide (2 mL), followed by addition of sodium bicarbonate (21.5 mg, 0.256 mmol) and benzyl bromide (8 μL, 0.067 mmol). After stirring for 24 hours at room temperature, deionized water (15 mL) was added and the reaction mixture was extracted by ethyl acetate (3 × 15 mL). The combined organic extracts were dried over MgSO4, filtered and concentrated *via* a rotary evaporator to yield a dark red oil. RP-HPLC purification on a C18 column with an acetonitrile-water gradient afforded cel-D7 as an orange solid (14.0 mg, 57.4% yield), with m/z 541.3306 (M+H).

Detailed characterization data of cel-D2 and cel-D7 are provided in Supplementary Figs. 1-6.

### Culture of HCC cell lines and primary hepatocytes

Human HCC cell lines HepG2, Huh7, and Hep3B were maintained in Dulbecco's Modified Eagle's Medium (DMEM) supplemented with 10% fetal bovine serum (Invitrogen, Carlsbad, CA), 100 μg/ml penicillin, and 100 μg/ml streptomycin. Cells were cultured at 37°C in a humidified atmosphere with 5% CO_2_. The HepG2 and Hep3B cell lines were obtained from American Type Culture Collection (Manassas, VA) in 2008. The Huh7 cell line was a gift from Dr. Mark Kay (Stanford University, CA) in 2003. All cell lines were last authenticated in June 2013, by the short tandem repeat profiling method at the Johns Hopkins Genetic Resources Core Facility. And all cell lines used were tested regularly for mycoplasma contamination.

Cryopreserved normal human hepatocytes and all special media and plates needed for their culture were received from CellzDirect/Invitrogen (Durham, NC). Characteristics of the three hepatocyte lots are shown in Supplementary Table 2A. Thawing and culture of primary hepatocytes were as described previously [47].

### Protein extraction, Western blotting, and co-immunoprecipitation

Total protein was extracted from tissues or harvested cells using T-PER Tissue Protein Extraction Reagent (Pierce; Rockford, IL), and protein concentration was determined with BCA Protein Assay Kit (Pierce, Rockford, IL). Equal amounts of protein (10 μg) were electrophoresed on 4% to 12% polyacrylamide gels (Invitrogen; Carlsbad, CA), and transferred onto polyvinylidene difluoride membranes, blocked with 10% nonfat milk for at least 1 hour, and probed with primary antibodies against CDC37 (ab61773) from Abcam (Cambridge, MA); HSP90 (SC-59577), B-Raf (SC-166), GAPDH (SC-365062) from Santa Cruz Biotechnology (Santa Cruz, CA); CDK4 (2906), EGFR (2232), MEK1/2 (9122), AKT (9272), A-Raf (4432), C-Raf (9422), p-A-Raf (s299, 4431), p-B-Raf (s445,2696), p-C-Raf (s289/296/301, 9431), p-AKT (s473, 4058s), p-MEK1/2 (s217/221, 9121) from Cell Signaling (Danvers, MA). The specific proteins were detected with HRP-conjugated secondary antibodies (Santa Cruz Biotechnology; Santa Cruz, CA) and SuperSignal West Pico or West Femto Maximum Sensitivity substrate from Pierce (Rockford, IL). Co-immunoprecipitation was performed using Protein A/G agarose (SC-2003, Santa Cruz Biotechnology, Santa Cruz, CA) according to manufacturer's instructions, with anti-CDC37 antibody (ab61773, Abcam, Cambridge, MA) for pull-down, and anti-HSP90 antibody (SC-59577, Santa Cruz Biotechnology, Santa Cruz, CA) for immunoblotting.

### Cell viability assay

Test compounds were added at desired final concentrations, and further incubated for 72 hours before cell viability was assessed using CellTiter-Glo Luminescent Cell Viability Assay (Promega; Madison, Wisconsin) as previously described [47]. The 50% inhibitory concentrations (IC_50_s; concentration of drug that inhibits cell growth by 50%) were calculated as an estimate of the cytotoxic effects of the four compounds. Three independent experiments were done, each in triplicates.

### Apoptosis analysis

Terminal dUTP-mediated nick-end labeling (TUNEL) assays (Promega, Madison, WI, USA) were performed according to the manufacturer's protocol. Briefly, HepG2 cells were seeded in 8-chamber BD tissue culture slides (BD Bioscience Labware, Bedmord, MA) at 10% confluency. Celastrol or its derivatives were added to the culture medium at final concentrations of 5 μM. After 6 hours incubation, cells were washed twice with PBS, and then fixed in 4% paraformaldehyde for 25 minutes. Fixed cells were washed twice in PBS with 0.1% Triton X-100, and then incubated with TUNEL reaction mixture for 60 minutes at 37°C. After washing with 2xSSC, slides were immersed in PBS (with 5 μg/ml DAPI) for 5 minutes in the dark, and then washed with PBS. Fluorescence labeling was visualized and photographed (100x magnification) with a fluorescence microscope (Nikon Eclipse 80i, Nikon Corporation, Tokyo, Japan) and with a digital camera (Nikon DXM1200f, Nikon Corporation, Tokyo, Japan). For TUNEL staining of the patient-derived xenografts, 6-μm tissue sections were stained using the ApopTag Peroxidase in Situ Oligo Ligation Apoptosis Detection Kit (Chemicon International, Temecula, CA) according to the manufacturer's protocol.

### Establishment of orthotopic HCC patient-derived xenografts

HCC tissues were collected from HCC patients who had undergone liver resection as part of their treatment. This study was approved by the Institutional Review Board at Stanford University for the use of human subjects in medical research, and informed consent was obtained from each patient prior to liver resection. Animal studies were carried out in compliance with Federal and local institutional rules for the conduct of animal experiments.

Characteristics of the three HCC patients are shown in Supplementary Table 2B. HCC specimens were mechanically and enzymatically dissociated in HBSS containing 0.1% collagenase, 0.01% hyaluronidase and 0.002% deoxyribonuclease at 37°C to obtain single cell suspensions [48]. Cells were then passed through a 70-μm filter, centrifuged at 100 g for 10 minutes and resuspended in Freezing Medium (FBS containing 10% DMSO) for storage at -80°C overnight, and transferred to liquid nitrogen for long-term storage. Thawed cells were suspended in BEGM medium mixed with 50% Matrix Matrigel (Becton Dickinson; Franklin Lakes, NJ) and injected subcutaneously into 4 week old (20 g body weight), male NOD.Cg-Prkdc^scid^Il2rg^tm1Wjl^/SzJ (Nod-SCID-Gamma; NSG). Mating pairs of NSG mice were originally purchased from Jackson Laboratory (Bar Harbor, MA), and bred according to approved institutional protocols. Once the subcutaneous xeongrafts reached 1 cm in diameter, they were harvested for dissociation as described above. Single cell suspensions were then transduced with self-inactivating lentivirus carrying an ubiquitin promoter driving a trifusion reporter gene, which harbors a bioluminescence (Luc2), a fluorescence (egfp), and a positron emission tomography reporter gene (ttk) at a multiplicity of infection of 5 [49]. High titer lentiviral vectors were produced using a modified version of a previous protocol [50]. Tumor cells were stained with Pacific BlueTM anti-mouse CD45, Pacific BlueTM anti-mouse H-2Kd, and Pacific BlueTM anti-mouse CD31 (BioLegend; San Diego, CA). Stable expressors were isolated by sorting as eGFP positive and Pacific Blue negative cells performed on a BD FACSAria (Becton Dickinson; Franklin Lakes, NJ).

To generate orthotopic HCC patient-derived xenografts, single tumor cells labeled with luciferase gene were suspended in BEME medium containing 50% Matrix Matrigel, and then subcutaneously injected into 4–8 week old male NSG mice (20-25 g body weight). Tumor development was monitored daily. Once the subcutaneous xenograft reached 1 cm in diameter, it was removed and cut into 2 mm^3^ pieces and surgically implanted into the left lobe of the liver of another group of 6 weeks old NSG mice [49]. Tumor growth was monitored once a week using the Xenogen IVIS *in vivo* imaging system (Caliper Life Sciences, Hopkinton, CA). Firefly luciferase imaging was acquired with D-Luc saline solution (150 mg/Kg body weight) *via* intraperitoneal injection.

### Animal treatment with celastrol and its derivatives

Treatment with celastrol and its derivatives were initiated within 1 week after transplantation of the orthotopic patient-derived xenografts. Tumor-bearing mice were randomized into groups (n = 5 each) to be intravenously injected with saline only (control); celastrol (4 mg/Kg); pristimerin (1 mg/Kg); cel-D2 (8 mg/Kg); cel-D7 (8 mg/Kg) (each diluted in saline) three times per week. Tumor growth was monitored weekly using the Xenogen IVIS *in vivo* imaging system, and growth curves were plotted using average bioluminescence within each group. Body weight was also measured weekly. After 3 weeks treatment, the mice were sacrificed and the tumors and normal livers harvested. Tumor size was measured with digital calipers and tumor volume was calculated using the formula π/6 × larger diameter × [smaller diameter]^2^. Liver and tumor tissues were fixed in formalin and embedded with paraffin. The 6-μm tissue sections were stained with hematoxylin and eosin (H&E) for evaluation of cell morphology and apoptosis as described above.

### *In vivo* toxicity studies of celastrol and its derivatives

BALB/cJ mice (6-8 weeks old) were randomized into groups (n = 4 each) to be intravenously injected with saline only (control); celastrol (4 mg/Kg); pristimerin (1 mg/Kg); cel-D2 (8 mg/Kg); or cel-D7 (8 mg/Kg) (each diluted in saline) three times per week. At the end of the administration period, mice in each group were euthanized and their bodies and harvested organs were weighed. In addition, blood was collected for whole blood complete blood counts (CBC) and plasma chemistry analysis at the Stanford Animal Diagnostic Laboratory. The normal ranges of CBC and plasma chemistry panel are obtained from the Mouse Phenome Database of the Jackson Laboratory.

### Statistical analysis

Statistical analyses were done using the SPSS version 15.0 software package (SPSS, Inc, Chicago, IL). Statistical significance was determined by independent samples t-test. P < 0.05 and P < 0.01 were considered statistically significant and highly significant, respectively.

## SUPPLEMENTARY MATERIAL FIGURES AND TABLES


