# Supplementary material for: Novel celastrol derivatives inhibit the growth of hepatocellular carcinoma patient-derived xenografts
Source: Oncotarget. 2014 Jul 10;5(14):5819–31. doi: 10.18632/oncotarget.2171 (PMC4170594; doi:10.18632/oncotarget.2171)
Supplement: Supplementary file 1 [file oncotarget-05-5819-s001.pdf]

Novel celastrol derivatives inhibit the growth of hepatocellular carcinoma patient-derived xenografts

Supplimentary Material

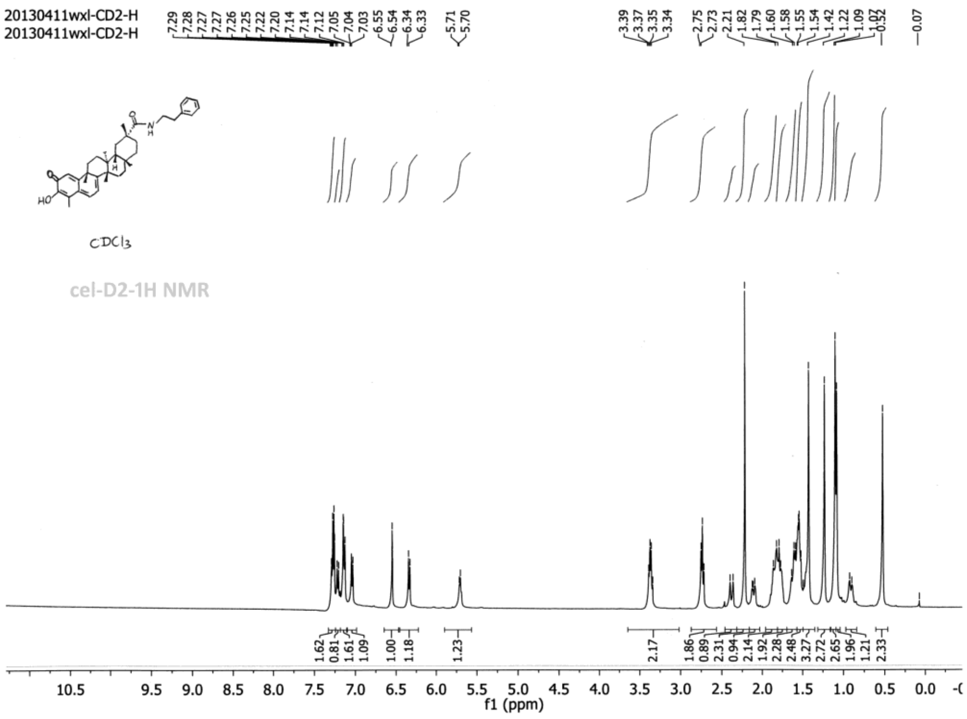

Supplementary Fig. 1: ^{1}H NMR data for cel-D2.

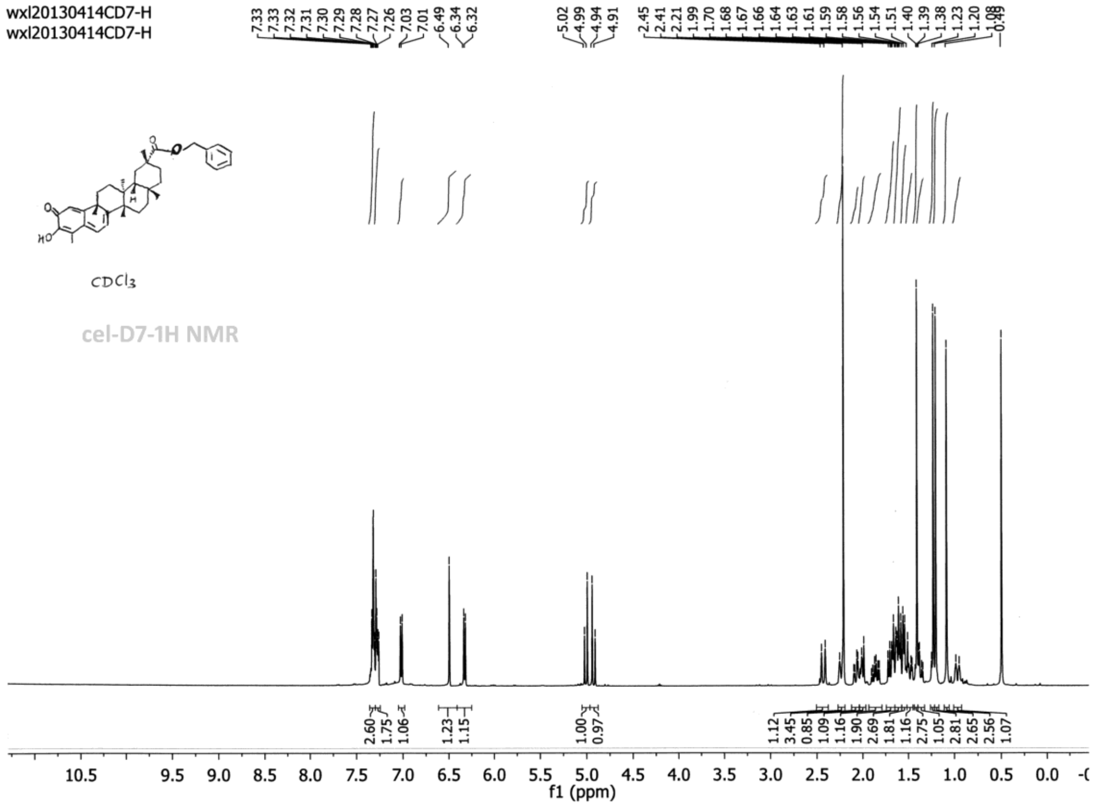

Supplementary Fig. 2: ^{1}H NMR data for cel-D7.

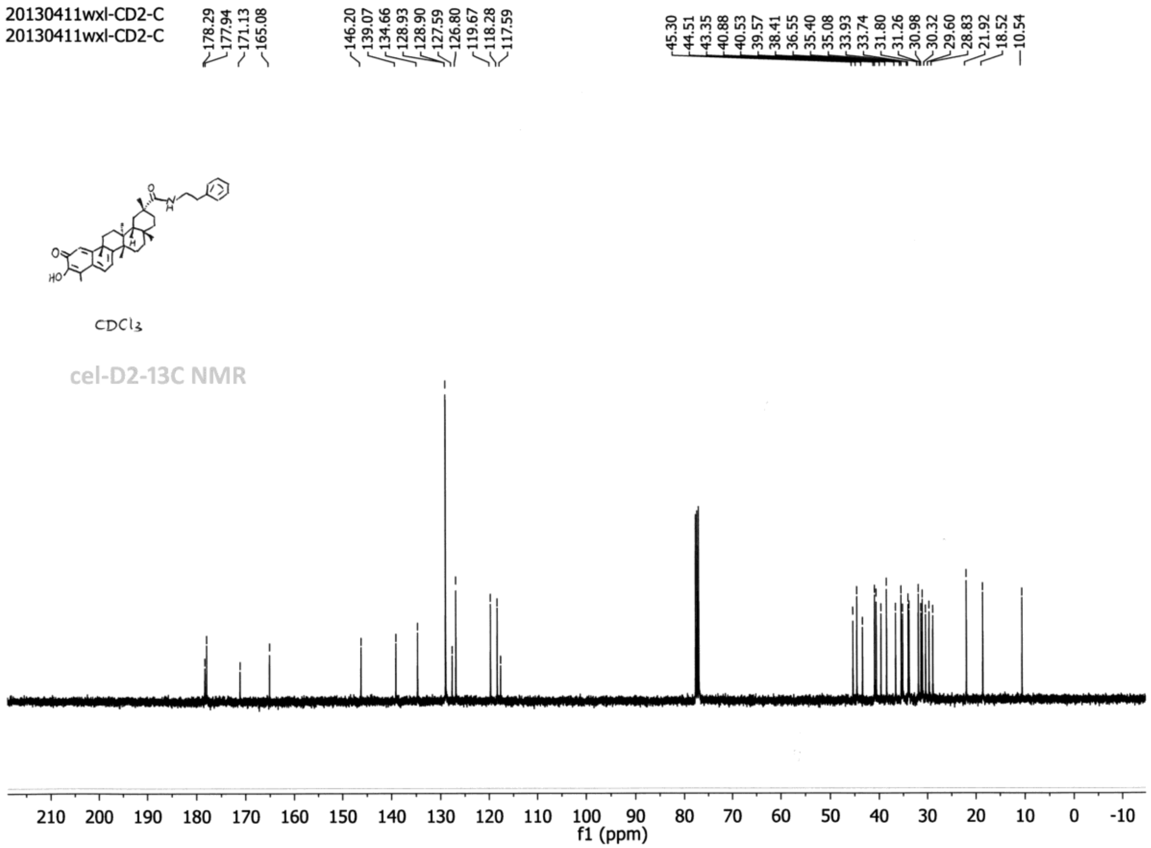

Supplementary Fig. 3: ^{13}C NMR data for cel-D2.

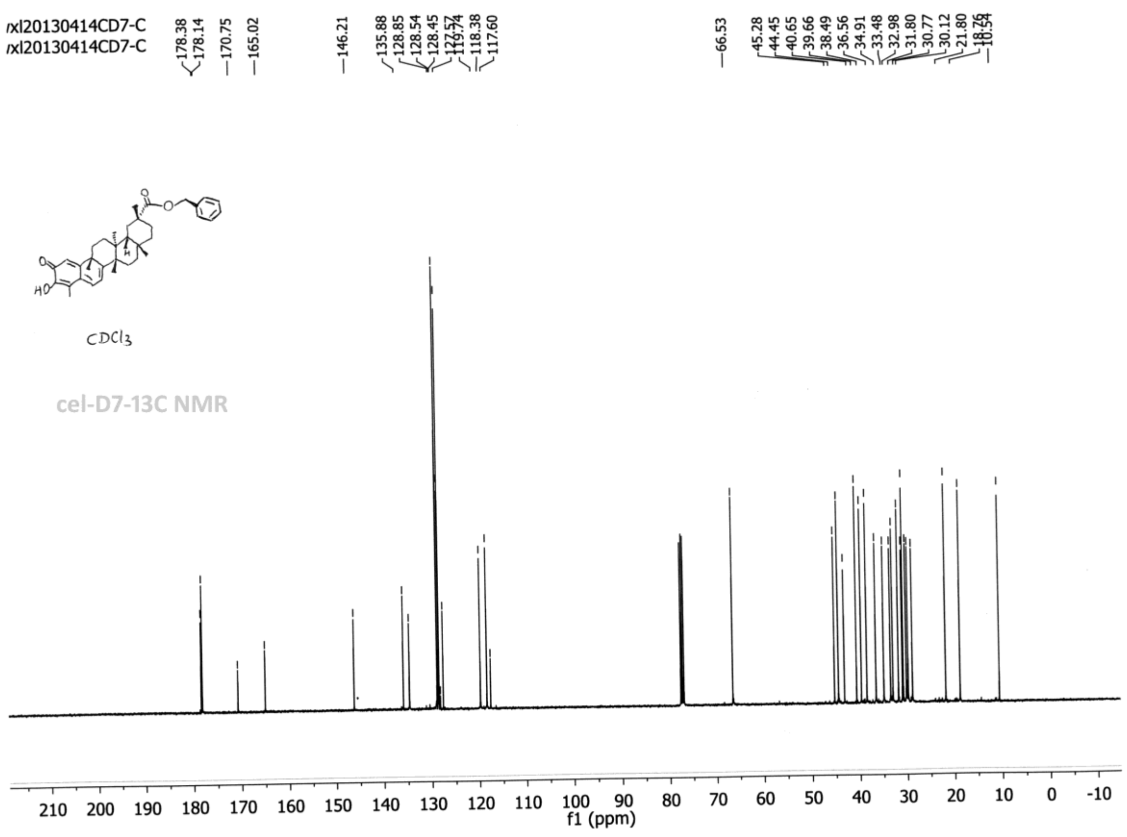

Supplementary Fig. 4: ^{13}C NMR data for cel-D7.

Mass Spectrum SmartFormula Report

Analysis Info

Analysis Name	D:\Data\HRAM\130415\data\WangX_41769_CD2_02_92_01_6857.d	Acquisition Date	4/15/2013 2:10:28 PM
Method	loopinj_pos_50_1200.m	Operator	BDAL@DE
Sample Name	WangX_41769_CD2_02	Instrument / Ser#	micrOTOF-Q II 10292
Comment			

Acquisition Parameter

Source Type	ESI	Ion Polarity	Positive	Set Nebulizer	2.5 Bar
Focus	Not active	Set Capillary	4500 V	Set Dry Heater	250 °C
Scan Begin	50 m/z	Set End Plate Offset	-500 V	Set Dry Gas	8.0 l/min
Scan End	1200 m/z	Set Collision Cell RF	100.0 Vpp	Set Divert Valve	Source

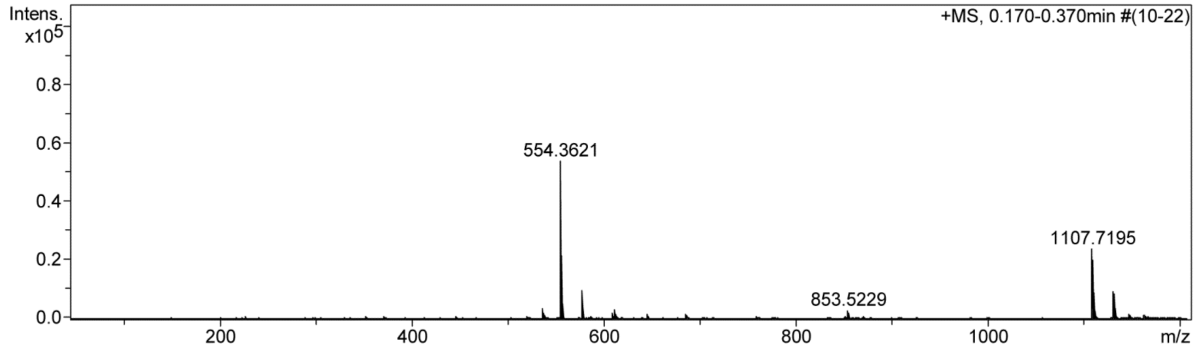

Meas. m/z	#	Formula	m/z	mSigma	err [ppm]	Score	err [mDa]	rdb	e¯ Conf	N-Rule
554.3621	1	C 37 H 48 N O 3	554.3629	4.9	1.4	100.00	0.8	14.5	even	ok
	2	C 35 H 49 N Na O 3	554.3605	11.2	-2.9	54.10	-1.6	11.5	even	ok
	3	C 33 H 44 N 7 O	554.3602	13.9	-3.4	42.09	-1.9	15.5	even	ok
	4	C 24 H 49 N 7 Na O 6	554.3637	69.1	2.8	11.27	1.6	3.5	even	ok
	5	C 21 H 41 N 17 Na	554.3623	70.1	0.4	22.93	0.2	9.5	even	ok
	6	C 22 H 44 N 13 O 4	554.3634	70.2	2.3	13.02	1.3	7.5	even	ok
	7	C 21 H 48 N 9 O 8	554.3620	72.6	-0.1	22.60	-0.0	2.5	even	ok
	8	C 20 H 45 N 13 Na O 4	554.3610	83.0	-2.0	8.80	-1.1	4.5	even	ok
	9	C 18 H 40 N 19 O 2	554.3607	84.1	-2.5	7.05	-1.4	8.5	even	ok

Supplementary Fig. 5: High resolution mass spectrometry data for cel-D2.

Mass Spectrum SmartFormula Report

Analysis Info

Analysis Name	D:\Data\HRAM\130415\data\WangX_41769_CD7_02_93_01_6858.d	Acquisition Date	4/15/2013 2:14:25 PM
Method	loopinj_pos_50_1200.m	Operator	BDAL@DE
Sample Name	WangX_41769_CD7_02	Instrument / Ser#	micrOTOF-Q II 10292
Comment			

Acquisition Parameter

Source Type	ESI	Ion Polarity	Positive	Set Nebulizer	2.5 Bar
Focus	Not active	Set Capillary	4500 V	Set Dry Heater	250 °C
Scan Begin	50 m/z	Set End Plate Offset	-500 V	Set Dry Gas	8.0 l/min
Scan End	1200 m/z	Set Collision Cell RF	100.0 Vpp	Set Divert Valve	Source

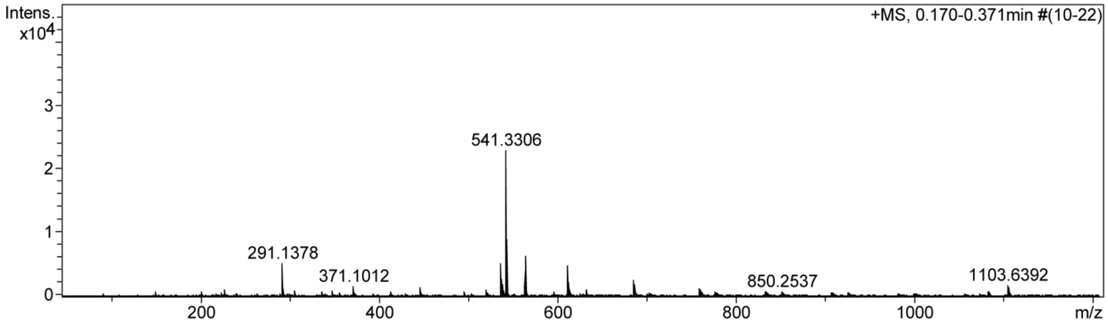

Meas. m/z	#	Formula	m/z	mSigma	err [ppm]	Score	err [mDa]	rdb	e¯ Conf	N-Rule
541.3306	1	C 36 H 45 O 4	541.3312	5.9	1.2	98.61	0.6	14.5	even	ok
	2	C 35 H 42 N 4 Na	541.3302	10.0	-0.8	100.00	-0.4	16.5	even	ok
	3	C 34 H 46 Na O 4	541.3288	11.1	-3.3	44.36	-1.8	11.5	even	ok
	4	C 32 H 41 N 6 O 2	541.3286	12.9	-3.8	34.73	-2.1	15.5	even	ok
	5	C 37 H 41 N 4	541.3326	16.9	3.6	34.66	2.0	19.5	even	ok
	6	C 23 H 46 N 6 Na O 7	541.3320	59.3	2.6	16.37	1.4	3.5	even	ok
	7	C 21 H 41 N 12 O 5	541.3317	69.2	2.1	13.74	1.1	7.5	even	ok
	8	C 20 H 38 N 16 Na O	541.3307	69.8	0.1	22.92	0.1	9.5	even	ok
	9	C 20 H 45 N 8 O 9	541.3304	71.6	-0.4	20.12	-0.2	2.5	even	ok
	10	C 19 H 42 N 12 Na O 5	541.3293	82.0	-2.4	7.62	-1.3	4.5	even	ok
	11	C 17 H 37 N 18 O 3	541.3291	83.1	-2.9	6.05	-1.6	8.5	even	ok

Supplementary Fig. 6: High resolution mass spectrometry data for cel-D7.

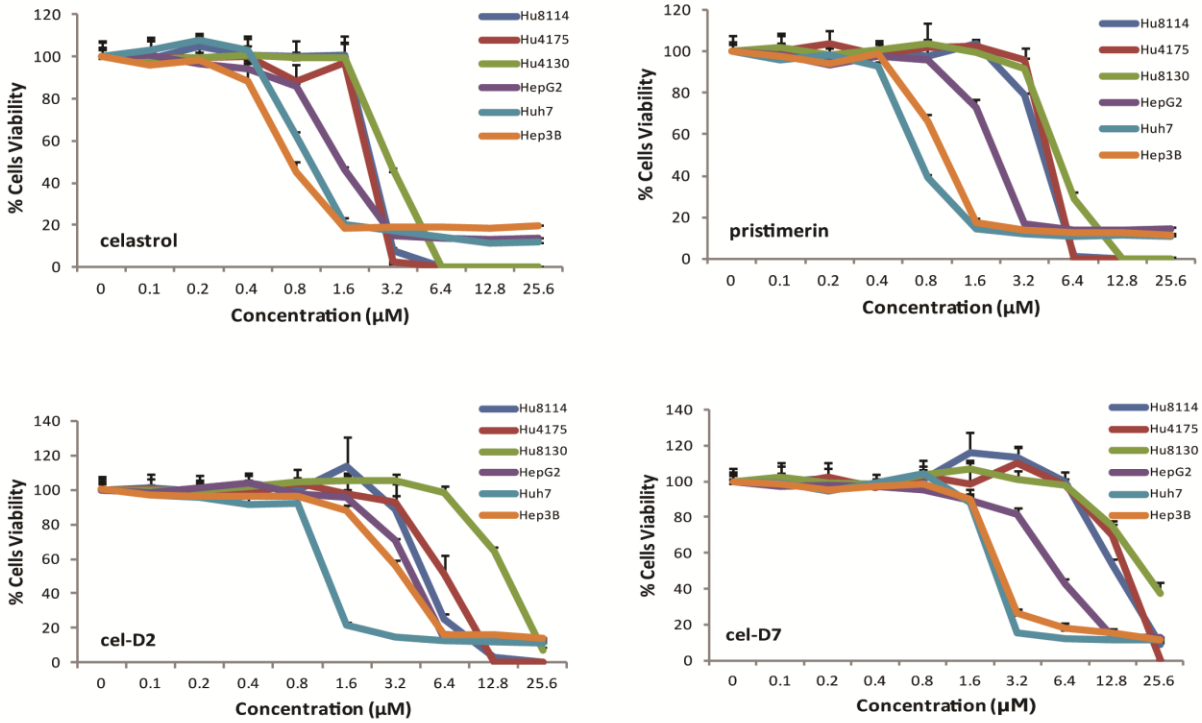

Supplementary Fig. 7: Celastrol and its derivatives preferentially inhibited viability of HCC cells compared to normal hepatocytes. Cell viability assays based on ATP release were used to determine the cytotoxicity of celastrol and its derivatives on three human HCC cell lines cells (HepG2, Huh7, and Hep3B) and normal hepatocytes (Hu8114, Hu4175, and Hu8130) following 72 hours of treatment. Results are presented as mean ± SD (error bars). Relative ATP activity is proportional to the number of viable cells. The values of luciferase activity were normalized and compared with the DMSO control value, which was set at 100% cell viability. Three independent experiments were done, each in triplicates.

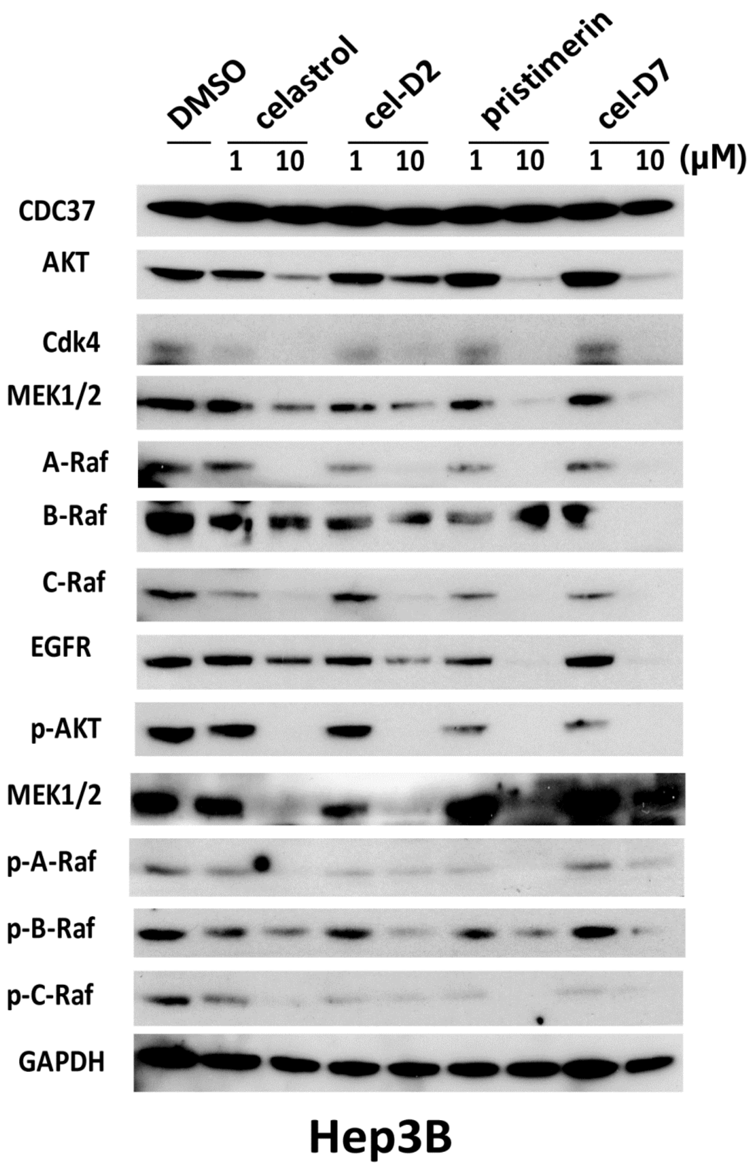

Supplementary Fig. 8: Celastrol and its derivatives induced degradation and inhibited phosphorylation of HSP90/CDC37 protein kinases in Hep3B cells. Hep3B cells were incubated for 6 hours with the compounds (1 or 10 μM) and CDC37, HSP90/CDC37 client proteins and GAPDH protein (loading control) levels were determined by Western blotting using specific antibodies.

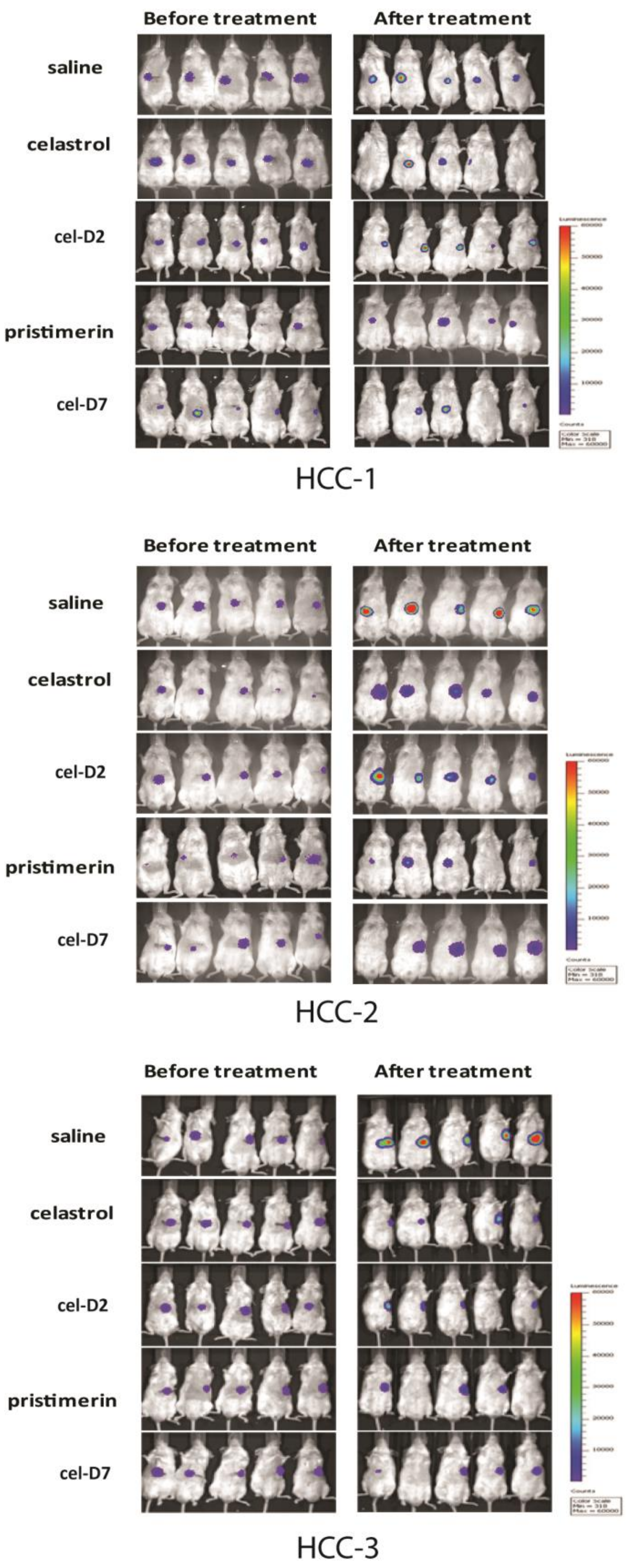

Supplementary Fig. 9: Bioluminescence images of three HCC patient-derived xenograft models are shown before treatment and after 3 weeks of treatment with celastrol or its derivatives. Orthotopic liver tumor models derived from human HCC patient specimens (HCC-1, HCC-2, HCC-3) expressing a trifusion

reporter gene were given intravenous injection of celastrol or its derivatives, and the tumor growth was monitored weekly using the Xenogen IVIS 100 imaging system.

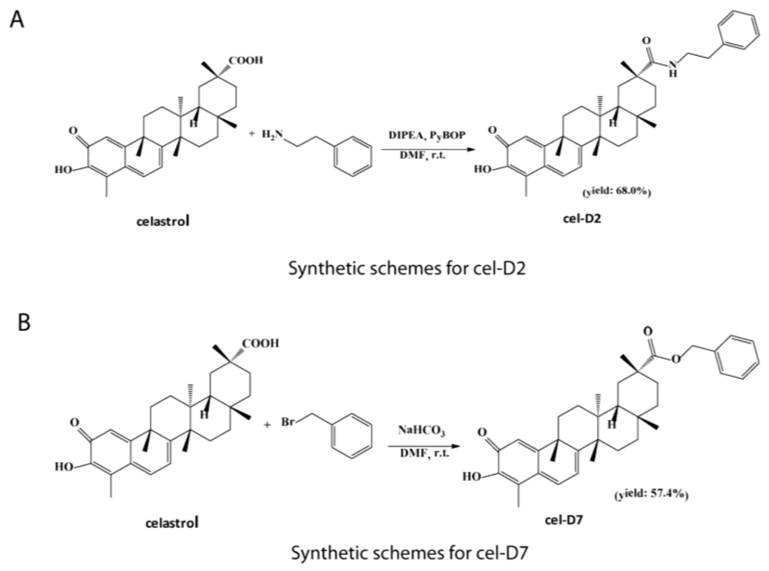

Supplementary Fig. 10: Synthetic schemes for (A) cel-D2 and (B) cel-D7.

Supplementary Table 1: Structure and activity of celastrol and its derivatives

Compound	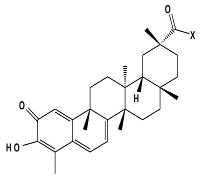 X	IC$_{50}$ to HCC cell lines (μM)	IC$_{50}$ to normal hepatocytes (μM)
celastrol	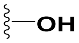	0.3-1.22	2.06-3.08
cel-D1	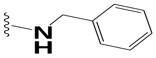	0.59-1.7	0.88-2.45
cel-D2	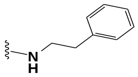	1.04-3.58	5.9-16.8
cel-D3	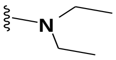	3.59-4.89	8.91-9.35
cel-D4	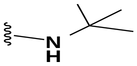	3.13-4.67	4.19-7.1
cel-D5	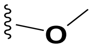	0.68-1.7	3.87-5.33
cel-D6	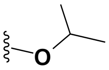	0.44-1.19	2.03-3.19
cel-D7	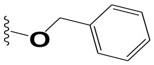	2.15-4.26	15.66-23.95

Supplementary Table 2A: Characteristics of normal hepatocytes

Lot	Age (y)	Gender	Race	Cell viability
Hu4175	3	Male	Caucasian	79%
Hu8114	47	Female	Caucasian	95%
Hu8130	18	Female	Caucasian	92%

Supplementary Table 2B: Characteristics of HCC patients

	Age (y)	Gender	Race	Virus
HCC-1	44	Male	Asian	HBV
HCC-2	75	Male	Caucasian	None
HCC-3	35	Male	Asian	HBV

Supplementary Table 3A: Selected organ weights after treatment in vivo

Organ	saline	celastrol	pristimerin	cel-D2	cel-D7
Brain	0.41±0.03	0.4±0.01	0.39±0.02	0.42±0.01	0.41±0.01
Heart	0.12±0.01	0.13±0.01	0.12±0.01	0.12±0.01	0.13±0.01
Kidney	0.39±0.03	0.37±0.02	0.36±0.04	0.4±0.02	0.39±0.03
Liver	1.1±0.06	1.14±0.07	0.98±0.09	1.13±0.09	1.05±0.13
Lung	0.17±0.02	0.17±0.01	0.15±0.01	0.18±0.01	0.17±0.02
Spleen	0.11±0.01	**0.19±0.03***	0.11±0.01	0.12±0.01	0.12±0.02
Body weight	24.17±0.73	**21.42±0.81***	**20.35±0.82***	23.85±1.43	22.83±0.97

Total body weights and selected organ weights of BALB/cJ mice (n=4/group) treated with different compounds. Data represent mean ± SD. (* $p < 0.05\%$, compared to saline group.)

Supplementary Table 3B: Selected toxicity results after treatment *in vivo*

Selected Parameters	saline	celastrol	pristimerin	cel-D2	cel-D7	Normal Range
WBC (K/µL)	6.34±2.34	**16.32±3.23***	4.26±1.49	4.04±2.09	4.49±2.21	3.2-12.8
RBC (M/µL)	9.58±0.55	9.17±0.50	8.23±0.89	6.96±2.44	7.23±2.41	8.71-11.6
HGB (g/dL)	14.83±0.49	13.96±0.39	12.70±1.36	11.10±3.03	11.98±2.35	13-17.2
Albumin (g/dL)	2.78±0.24	2.78±0.05	2.93±0.28	2.56±0.15	2.55±0.13	2.5-2.9

Creatine (mg/dL)	0.35 ±0.10	0.45±0.06	0.15±0.06	0.28±0.09	0.38±0.10	0.25-0.47
ALP(IU/L)	89.25±29.92	48.75±4.50	103.8±26.52	78.00±19.70	92.50±9.68	82-172
AST(U/L)	201.8±60.07	169.0±94.63	**804.8±753.8***	207.75±62.45	171.3±84.0	57-382
ALT(U/L)	145.0±63.69	85.75±68.09	**475.8±266.4***	193.8±115.7	92.00±94.08	21-187
Total Bilirubin (mg/dL)	0.05 ±0.10	0.15±0.10	0.00±0.00	0.00±0.00	0.15±0.24	0.03-0.23
Total protein (g/dL)	5.55±0.68	5.70±0.00	5.35±0.34	5.13±0.17	4.85±0.17	4.4-5.9
BUN (mg/dL)	22.00±5.60	19.50±1.73	18.75±2.50	18.50±1.29	18.00±0.82	17-30

Representative toxicological data of BALB/cJ mice (n=4/group) treated with different compounds. WBC: white blood cells; RBC: red blood cells; HGB: hemoglobin; ALP: alkaline phosphatase; AST: aspartate aminotransferase; ALT: alanine aminotransferase; BUN: blood urea nitrogen. Data represent mean ± SD. (* $p < 0.05\%$, compared to saline group.)
